# Solution-Plasma Synthesis and Characterization of Transition Metals and N-Containing Carbon–Carbon Nanotube Composites

**DOI:** 10.3390/ma17020320

**Published:** 2024-01-08

**Authors:** Kodai Sasaki, Kaiki Yamamoto, Masaki Narahara, Yushi Takabe, Sangwoo Chae, Gasidit Panomsuwan, Takahiro Ishizaki

**Affiliations:** 1 Materials Science and Engineering, Graduate School of Engineering and Science, Shibaura Institute of Technology, 3-7-5 Toyosu, Koto-ku, Tokyo 135-8548, Japan; mb22019@shibaura-it.ac.jp (K.S.); mb22047@shibaura-it.ac.jp (K.Y.); mb22034@shibaura-it.ac.jp (M.N.); mb23025@shibaura-it.ac.jp (Y.T.); 2 SIT Research Laboratories, Shibaura Institute of Technology, 3-7-5 Toyosu, Koto-ku, Tokyo 135-8548, Japan; chae@sic.shibaura-it.ac.jp; 3 Department of Materials Engineering, Faculty of Engineering, Kasetsart University, Bangkok 10900, Thailand; gasidit.p@ku.ac.th; 4 Department of Materials Science and Engineering, College of Engineering, Shibaura Institute of Technology, 3-7-5 Toyosu, Koto-ku, Tokyo 135-8548, Japan

**Keywords:** solution plasma, ORR catalyst, carbon nanotubes, composite materials

## Abstract

Lithium–air batteries (LABs) have a theoretically high energy density. However, LABs have some issues, such as low energy efficiency, short life cycle, and high overpotential in charge–discharge cycles. To solve these issues electrocatalytic materials were developed for oxygen reduction reaction (ORR) and oxygen evolution reaction (OER), which significantly affect battery performance. In this study, we aimed to synthesize electrocatalytic N-doped carbon-based composite materials with solution plasma (SP) using Co or Ni as electrodes from organic solvents containing cup-stacked carbon nanotubes (CSCNTs), iron (II) phthalocyanine (FePc), and N-nethyl-2-pyrrolidinone (NMP). The synthesized N-doped carbon-based composite materials were characterized by transmission electron microscopy (TEM), X-ray diffraction (XRD), Raman spectroscopy, and X-ray photoelectron spectroscopy (XPS). TEM observation and XPS measurements revealed that the synthesized carbon materials contained elemental N, Fe, and electrode-derived Co or Ni, leading to the successful synthesis of N-doped carbon-based composite materials. The electrocatalytic activity for ORR of the synthesized carbon-based composite materials was also evaluated using electrochemical measurements. The electrochemical measurements demonstrated that the electrocatalytic performance for ORR of N-doped carbon-based composite material including Fe and Co showed superiority to that of N-doped carbon-based composite material including Fe and Ni. The difference in the electrocatalytic performance for ORR is discussed regarding the difference in the specific surface area and the presence ratio of chemical bonding species.

## 1. Introduction

Metal–air batteries (MABs) have a theoretically high energy density because they utilize atmospheric oxygen as the active cathode material, which increases the proportion of metal that acts as the active anode material [1,2,3,4]. The oxygen reduction reaction (ORR) and oxygen evolution reaction (OER) occur at the cathode during the discharging and charging of MABs, and the efficacy of these reactions significantly impacts the battery performance. Improving the cycle performance during the charging–discharging of MABs is essential for their practical applications; thus, the development of catalyst materials that increase the efficiency of the ORR and OER is required. Typical catalyst materials for the ORR and OER include platinum and ruthenium. However, these are precious metals with limited reserves and high costs; thus, there is a need for the development of alternative materials.

Carbon nanotubes (CNTs) are suitable as electrode materials due to their electrical conductivity [5]. However, because of the low ORR activity of CNTs, their catalytic activity for the ORR must be improved. This can be achieved by doping to CNTs with heteroatoms such as nitrogen. It has been reported that ORR performance is greatly improved when carbon doped with heteroatoms is combined with macrocyclic metal complexes such as metal phthalocyanines and porphyrins [6,7,8,9,10]. The catalytic performance for the ORR can also be improved by incorporating transition metal nanoparticles into carbon doped with heteroatoms [11,12].

One method of doping a material with heteroatoms or incorporating transition metals is the solution-plasma (SP) process. SP is a non-thermal-equilibrium, low-temperature plasma that is generated in bubbles in the liquid phase while they are being formed between the electrodes when a high voltage is applied between opposing electrodes in a solution using a bipolar pulse power source. SP can form various active species. Because SP uses a low-temperature reaction field in solution, it enables the synthesis of materials that cannot be easily synthesized with gas-phase plasma or high-temperature plasma in a liquid [8,9,10,11,13,14,15,16,17,18,19,20,21,22,23,24,25,26,27,28,29,30,31,32,33]. SP treatment in an organic solvent containing heteroatoms enables the synthesis of carbon materials in a bottom-up manner while incorporating the heteroatoms into the carbon material. In addition, SP can synthesize composite materials in one step, reducing the cost and energy required for synthesis compared to other processes. Hyun et al. synthesized N-doped carbon nanosheets using SP treatment at high frequency with N-methyl-2-pyrrolidinone (NMP) as a solvent [21,22]. The ORR performance can also be improved by adding iron (II) phthalocyanine (FePc) to the organic solvent to introduce Fe-N bonds into carbon, which effectively improves the ORR activity [23]. It is also possible to form composites of CNTs with carbon doped with heteroatoms by dispersing the conductive CNTs in an organic solvent and subjecting them to SP treatment [8,13,24]. The resulting composite materials exhibit excellent catalytic properties for the ORR [8,13]. This enhancement is plausibly due to the synergistic effect of the high conductivity of the CNTs and the introduction of ORR active bonding species, such as graphitic N and pyridinic N, into the synthesized carbon and CNTs. Therefore, superior catalytic performance can be achieved by combining FePc with a composite of CNTs and N-doped carbon.

Moreover, the SP can form electrode-derived nanoparticles and nanoclusters, as it can sputter electrode-derived metals [25,26,27,28,29,30,31,32,33]. Thus, Co and Ni nanoparticles can be formed using Co and Ni as the SP electrodes. These metal nanoparticles are used as catalysts for the synthesis of single-walled carbon nanotubes (SWNTs). Using the laser-oven method, Smalley et al., for the first time, successfully synthesized a large quantity of SWNTs by adding approximately 1 at% of microparticulate Co and Ni to the raw carbon material as catalysts [34]. Therefore, by using Co and Ni as SP electrodes, Co and Ni nanoparticles can be sputtered in an organic solvent, resulting in the formation of Co or Ni nanoparticles. The produced nanoparticles act as catalysts in the synthesis of carbon materials with high crystallinity. In addition, the nanoparticles can be introduced into the synthesized carbon materials. This introduction of Co or Ni nanoparticles into the carbon materials can improve the electrocatalytic activity of the synthesized carbon materials for the ORR. Therefore, these nanoparticles can prospectively be used in the synthesis of carbon materials with favorable crystallinity.

In this study, FePc and cup-stacked carbon nanotubes (CSCNTs) were dispersed in the organic solvent NMP and subjected to SP treatment using cylindrical Co and Ni rods as electrodes. This process synthesized carbon-based composite materials of CSCNTs and N-containing carbon (containing Fe-N bonds and electrode-derived Co or Ni nanoparticles). The physicochemical properties of the synthesized materials and their electrocatalytic performance for the ORR were investigated.

## 2. Materials and Methods

### 2.1. Synthesis of Carbon Materials

**Figure 1:**
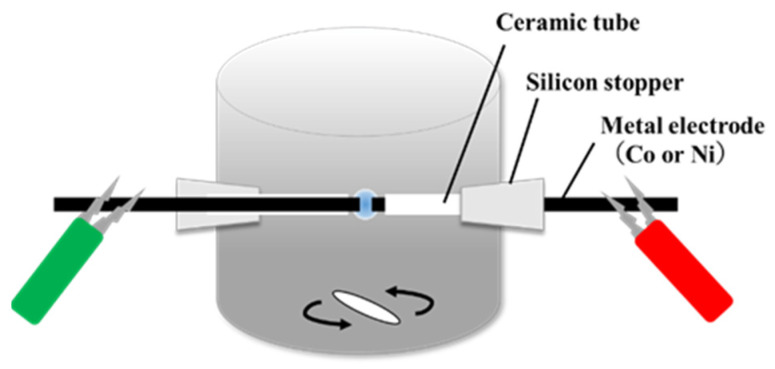
Schematic illustration of solution plasma equipment.

First, 17 mg of FePc (Tokyo Chemical Industry Co., Tokyo, Japan, >97.0%) and 50 mg of CSCNTs (GSI Creos Corp., Carbere, Tokyo, Japan) were ultrasonically dispersed in 100 mL of NMP (Kanto Chemical Co., Tokyo, Japan, >99.0%). The mixed solution was used as raw material for the synthesis of carbon materials. A cobalt wire (Co wire, Nilaco Corp., Tokyo, Japan, >99.995%) or nickel wire (Ni wire, Nilaco Corp., Tokyo, Japan, >99%) with a diameter of 1.0 mm was covered with an insulating ceramic tube, and the two pieces of Co or Ni wire were placed at the center of a glass reactor with a gap distance of 1 mm for the SP treatment. The Co or Ni wire was sputtered during SP treatment, resulting in the formation of Co or Ni nanoparticles. The distance between the electrodes was adjusted to 1.0 mm. Plasma was generated in the mixed solution at room temperature and atmospheric pressure with an applied voltage of 1.0 kV, frequency of 200 kHz, and pulse width of 1.0 µsec using a bipolar pulsed power supply (MPP-HV04, Kurita Seisakusho Co., Ltd., Kyoto, Japan). The SP treatment time was 10 min, and all SP processes were performed in a draft chamber because electrode material or organic solvent-derived vapor could be generated during synthesis. Figure 1 presents a schematic of the SP process. After the SP treatment, the solution was suction-filtered using a 0.1 µm polytetrafluoroethylene (PTFE) membrane filter (JVWP04700, Merck Millipore, Burlington, MA, USA) for several durations. The filtrate was washed with ethanol and acetone several times, and after suction filtration, the filtrate was dried in an electric furnace at 200 °C for 1 h, which yielded the carbon sample. Hereinafter, the carbon materials synthesized using Co and Ni wire are referred to as Co-NC@CNT and Ni-NC@CNT, respectively.

### 2.2. Evaluation of the Synthesized Carbon Materials

The crystal structures of the synthesized samples were determined using an X-ray diffractometer (XRD, Smart Lab, Rigaku, Tokyo, Japan). XRD measurements were carried out using CuKα X-rays within a 2θ range of 5–90° at a scanning speed of 10.0°/min. X-ray photoelectron spectroscopy (XPS, JPS-9010MC, JEOL Ltd., Tokyo, Japan) was used to analyze the surface elemental composition and binding state. XPS measurements were carried out using MgKα X-rays at a voltage of 10 kV and a current of 25 mA. The specific surface area of the samples was measured using an automatic specific surface area/pore distribution measurement device (Tristar II 3020, Shimadzu, Kyoto, Japan). The samples were degassed at 100 °C for 24 h as a pretreatment. The crystallinity of the synthesized carbon was evaluated using a Raman spectrometer (NRS-5100, JEOL Ltd., Tokyo, Japan). Raman spectra were acquired using a laser with an excitation wavelength of 532.5 nm in the wavenumber range of 100–3000 cm^−1^ with an exposure time of 10 s and an integration count of five. Morphological observation and elemental analysis of the synthesized carbon materials were performed using a transmission electron microscope (TEM, JEM-2100, JEOL, Ltd., Tokyo, Japan) equipped with an energy-dispersive X-ray spectroscope (EDS, JED-2300, JEOL, Ltd., Tokyo, Japan). The accelerating voltage was set to 200 kV.

### 2.3. Electrochemical Measurements

Using the synthesized samples as active electrode materials, the electrocatalytic activity for the ORR was evaluated using a dual electrochemical analyzer (ALS704ES, BAS). The carbon material (5 mg) was added to a mixed solution consisting of 950 µL of 2-propanol (Kanto Chemical Co., Tokyo, Japan, >99.7%) and 50 µL of Nafion perfluorinated resin solution (Sigma-Aldrich, St. Louis, MI, USA), which was then dispersed by ultrasonic stirring for 10 min to prepare a slurry. The prepared slurry (7.5 µL) was added dropwise to a Pt-GC electrode, which was then dried under atmospheric pressure overnight and used as a working electrode. An Ag/AgCl electrode in saturated aqueous KCl solution and a Pt electrode were used as the reference and counter electrodes, respectively. A 0.1 M aqueous KOH solution was used as the electrolyte. Linear sweep voltammetry (LSV) and chronoamperometry (CA) measurements were performed in an oxygen-saturated environment using a rotating ring-disk electrode device (RRSE-3A, BAS, manufacturer, Tokyo, Japan). LSV data were acquired at a rotational speed of 1600 rpm and a scan speed of 10 mV/s in the scan range of 0 to −1 V vs. Ag/AgCl. In this study, all potentials were converted to the reversible hydrogen electrode (RHE) standard using the following equation:

*E*_RHE_ = *E*_Ag/AgCl_ + 0.197 + 0.0591 × pH

CA data were acquired for 20,000 s at a rotation speed of 1600 rpm and a potential of 0.45 V (vs. RHE).

## 3. Results and Discussion

### 3.1. Characterization of the Synthesized Carbon Materials

**Figure 2:**
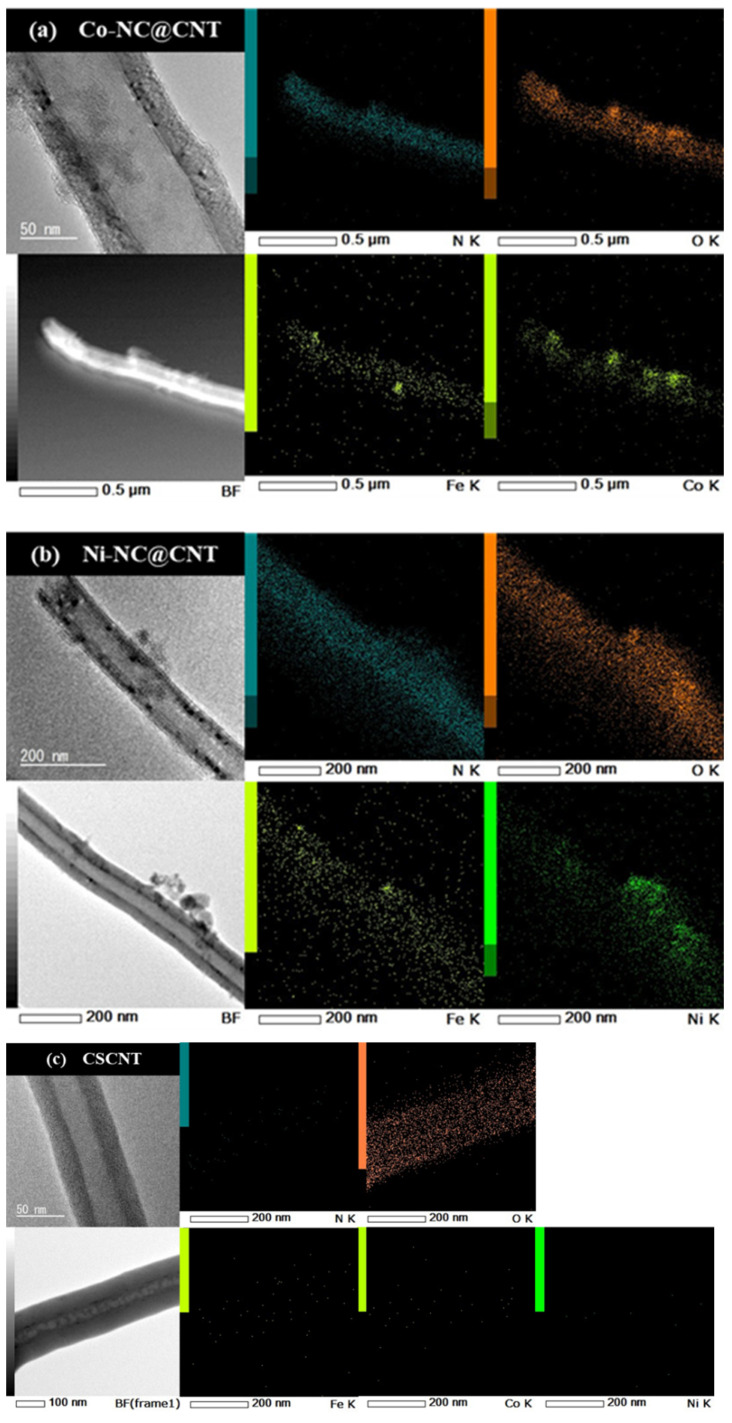
TEM and elemental mapping images of (**a**) Co-NC@CNT, (**b**) Ni-NC@CNT, and (**c**) pristine CNT.

Figure 2 shows the TEM and elemental mapping images of (a) Co-NC@CNT, (b) Ni-NC@CNT, and (c) pristine CNT. The TEM images of Co-NC@CNT and Ni-NC@CNT show the presence of thin, amorphous products on the CSCNT surface, whereas a hollow structure was observed in the TEM image of pristine CNT. The mapping images in Figure 2a,b show the uniform dispersion of N without aggregation, suggesting that the CSCNT surface was coated with N-containing carbon derived from NMP. On the other hand, in TEM and elemental mapping images of pristine CNT (Figure 2c), the presence of N was not confirmed in the elemental mapping images of CSCNT. As shown in Figure 2a,b, the EDS mapping images show the aggregation of Co and O, as well as Ni and O, in the same locations, indicating the possible formation of Co and Ni oxides. Furthermore, a small amount of FePc-derived Fe was observed in both Co-NC@CNT and Ni-NC@CNT, showing that these samples contained Fe. In contrast, the presence of Fe, Co, and Ni elements was not confirmed in pristine CNT. N and other elements did not exist in the pristine CSCNTs, and it was concluded that N, Fe, Co, and Ni were introduced in the SP process.

**Figure 3:**
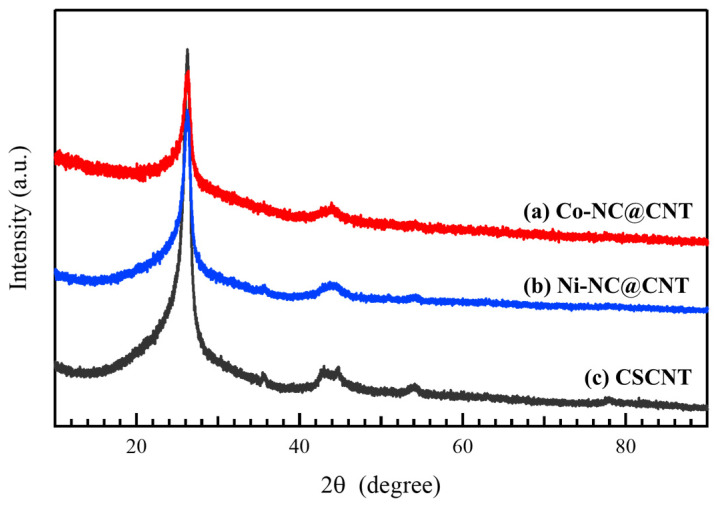
XRD patterns of (a) Co-NC@CNT, (b) Ni-NC@CNT, and (c) CSCNT.

Figure 3 shows the XRD patterns of (a) Co-NC@CNT, (b) Ni-NC@CNT, and (c) pristine CSCNT. Two peaks at around 2θ = 26° and 42° are attributed to the 002 and 110 reflections of the graphite structure contained in CSCNT, respectively, which were observed in the XRD patterns of all samples, demonstrating that the CSCNT structure was maintained and was not affected by the SP process. The peaks derived from Co oxides and Co observed by TEM were absent in the XRD patterns of Co-NC@CNT, suggesting that the Co and Co oxides were amorphous. Similarly, the absence of peaks derived from Ni or Ni oxides in the XRD patterns of Ni-NC@CNT suggests that these species were also amorphous. Because no Fe-derived peaks were observed in the XRD pattern of Co-NC@CNT or Ni-NC@CNT, it is likely that the content of Fe was low or that Fe was in an amorphous state.

**Figure 4:**
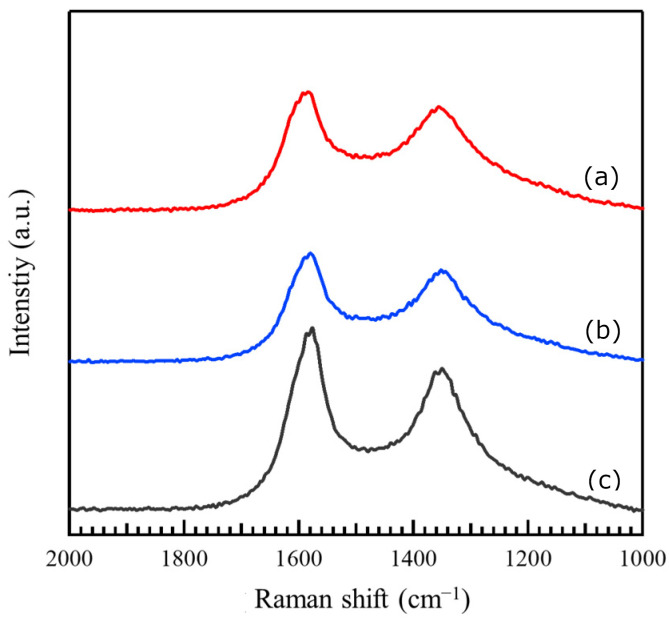
Raman spectra of (a) Co−NC@CNT, (b) Ni−NC@CNT, and (c) CSCNT.

Figure 4 shows the Raman spectra of (a) Co-NC@CNT, (b) Ni-NC@CNT, and (c) CSCNT, showing a peak at around 1360 cm^−1^ (D band) attributed to disorder or defects in the graphite structure and another peak at around 1580 cm^−1^ (G band) derived from the graphite structure. The relative intensity of these peaks (I_D_/I_G_) was used to evaluate the crystallinity of the carbon material. In general, the crystallinity of the carbon material is reduced with an increase in the I_D_/I_G_ ratio. The I_D_/I_G_ ratios of Co-NC@CNT, Ni-NC@CNT, and CSCNT were 0.731, 0.738, and 0.693, respectively. The SP-treated carbon materials had a higher I_D_/I_G_ ratio than untreated CSCNT, indicating that the crystallinity of the SP-treated carbon materials was lowered. This is due to the fact that the carbon materials coated on CSCNT by the SP treatment had lower crystallinity than CSCNT. The coated carbon materials contained NMP-derived N, electrode-derived Co or Ni, and FePc-derived Fe, which likely resulted in lower crystallinity compared to untreated CSCNT.

Table 1 shows the elemental composition of the samples obtained from the XPS measurements. Co−NC@CNT contained N, Co, and Fe, but the contents of these elements were low (<1 at%). Similarly, Ni-NC@CNT contained N, Ni, and Fe, and the contents of these elements were low. The existence ratio of N and Fe in the SP-treated carbon materials did not differ with the type of electrode used for SP.

**Figure 5:**
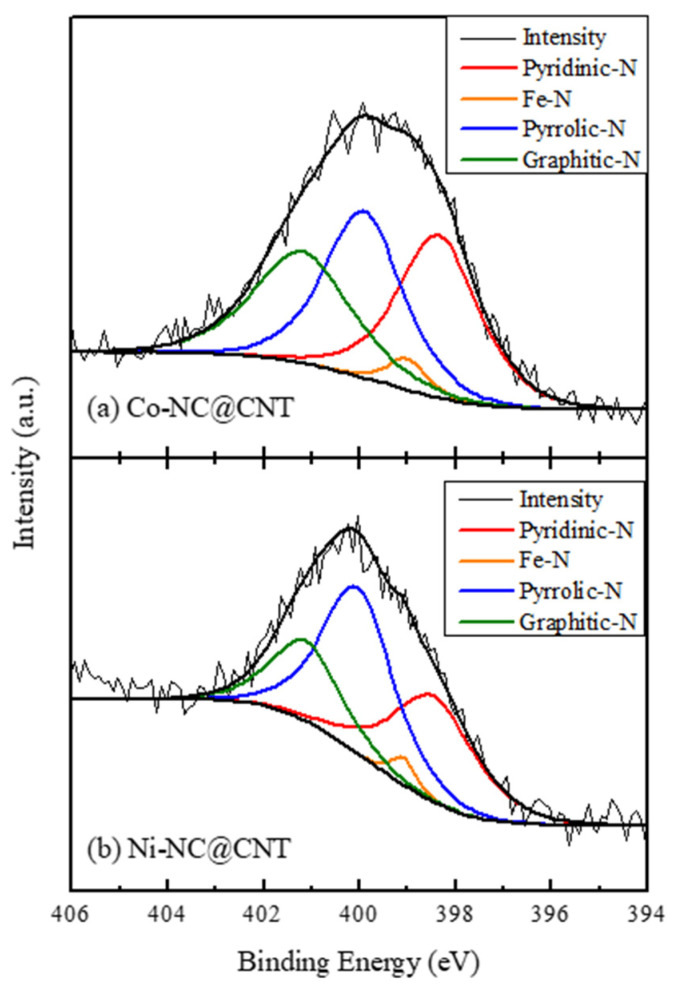
XPS N1*s* spectra of (**a**) Co-NC@CNT and (**b**) Ni-NC@CNT.

Figure 5 shows the deconvoluted N 1*s* XPS spectra of (a) Co-NC@CNT and (b) Ni-NC@CNT, and the ratios of nitrogen-bound species obtained from the deconvoluted spectra are listed in Table 2. As shown in Figure 5a,b, the N 1*s* spectra could be deconvoluted into four peaks assigned to pyridinic-N, Fe-N, pyrrolic N, and graphitic N at 398.4, 398.7, 400.0, and 401.2 eV, respectively, indicating the presence of these species. As indicated in Table 2, the ratio of pyrrolic N and graphitic N differed depending on the type of electrode used in the SP treatment. Compared with Ni-NC@CNT, Co-NC@CNT had higher ratios of graphitic N and Fe-N, which have been reported to be effective in improving the ORR activity. The surface area of a material is also an important factor that affects the electrocatalytic properties for the ORR. Thus, the specific surface area of each sample was calculated via BET measurements. The BET-specific surface area of Co-NC@CNT, Ni-NC@CNT, and CSCNT was estimated to be 75, 69, and 52 m^2^/g, respectively. The SP-treated carbon materials had a larger specific surface area than the untreated CSCNT, and Co-NC@CNT had a higher specific surface area than Ni-NC@CNT. Based on the abundance ratio of the nitrogen-bound species (graphitic N and Fe-N) effective in improving the ORR performance and the specific surface area, Co-NC@CNT is expected to exhibit superior ORR properties compared to Ni-NC@CNT.

### 3.2. Electrocatalytic Activity of the Synthesized Carbon Materials

**Figure 6:**
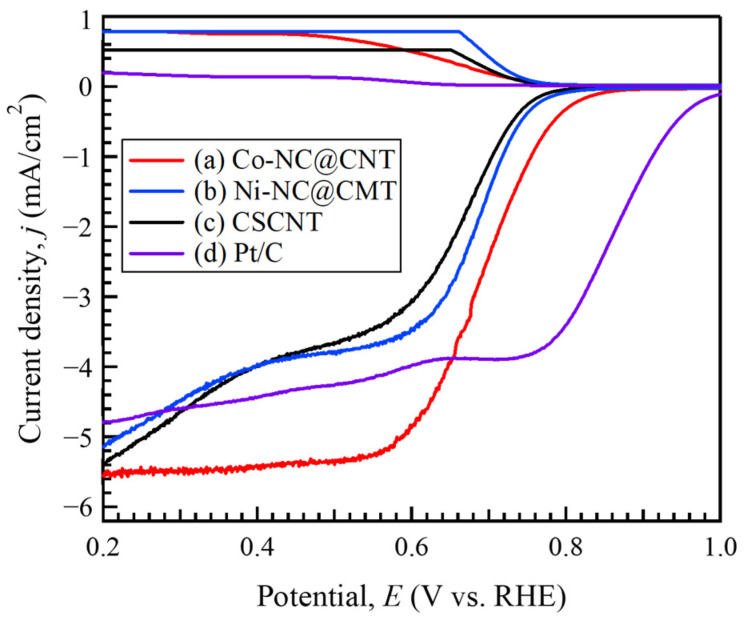
Linear sweep voltammograms (LSVs) of (a) Co-NC@CNT, (b) Ni-NC@CNT, (c) CSCNT, and (d) commercial Pt/C at 1600 rpm in the O_2_-saturated 0.1 M KOH solution.

Figure 6 shows the LSV curves of Co-NC@CNT, Ni-NC@CNT, and CSCNT. For comparison, the LSV curves of commercially available 20 wt.% Pt carbon (Pt/C) are also shown in Figure 6. The onset and half-wave potentials for the ORR of Co-NC@CNT, Ni-NC@CNT, CSCNT, and Pt/C were found to be 0.783 and 0.693, 0.749 and 0.686, 0.749 and 0.667, and 0.954 V and 0.860 V, respectively. The onset and half-wave potentials of the SP-treated CSCNTs exhibited a more notable positive shift than those of untreated CSCNT, leading to the improvement of the catalytic performance. However, the catalytic performance of the synthesized catalysts was inferior to that of Pt/C. The onset and half-wave potentials of Co-NC@CNT showed more notable changes than those of Ni-NC@CNT, which is likely due to the differences in the abundance ratios of the nitrogen-bound species in the synthesized carbon materials. The abundance ratios of graphitic N and Fe-N, which effectively improve the onset potential for the ORR, were higher in Co-NC@CNT than in Ni-NC@CNT, suggesting the superiority of Co-NC@CNT. In addition, the onset and half-wave potentials for the ORR of Co-NC@CNT, Ni-NC@CNT, and CSCNT were also compared to the reported carbon-based electrocatalytic materials to provide a clearer picture of the materials’ relative performance [6,8,10,12,13,20,22,23,29,35]. A comparison of ORR performance for various carbon-based electrocatalytic materials is shown in Table S1. As clearly seen in Table S1, the ORR performance of our samples treated by the SP process was inferior to the that of the reported carbon-based electrocatalytic materials. This could be due to the fact that the doping amounts of elemental metal (Fe, Co, or Ni) and N in our synthesized carbon samples were lower than those of the reported carbon-based electrocatalytic materials. Therefore, an increase in the doping amounts of elemental metal (Fe, Co, or Ni) and N to carbon materials in the SP process would be required to improve the ORR performance. To realize this, further study will be required.

The limiting current density of Co-NC@CNT, Ni-NC@CNT, CSCNT, and Pt/C at 0.45 V (vs. RHE) was found to be 5.36, 3.87, 3.81, and 4.33 mA/cm^2^, respectively. The limiting current density of Co-NC@CNT showed a greater improvement relative to that of CSCNT and was greater than that of Pt/C, indicating that the catalytic activity of Co-NC@CNT is superior to that of Pt/C. Additionally, Co-NC@CNT had a considerably higher limiting current density than Ni-NC@CNT. Because the specific surface area of Co-NC@CNT was greater than that of Ni-NC@CNT, Co-NC@CNT was expected to have a larger number of catalytically active sites. Moreover, the existence ratio of the pyridinic-N and graphitic N bond species in Co-NC@CNT was higher than that of Ni-NC@CNT. Zheng et al. reported that pyridinic-N and graphitic N affected the improvement of electrocatalytic properties for the ORR of carbon materials [36]. Thus, the electrocatalytic properties of Co-NC@CNT were higher than those of Ni-NC@CNT. 

**Figure 7:**
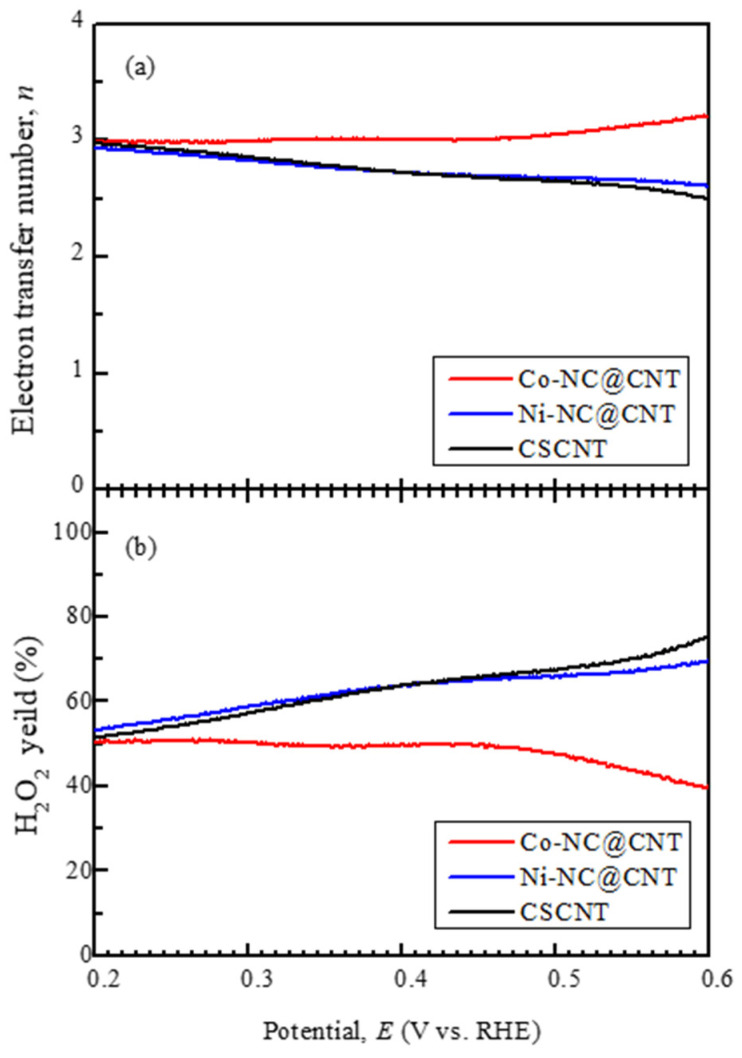
(**a**) Change in electron transfer numbers of Co-NC@CNT, Ni-NC@CNT, and CSCNT as a function of potentials. (**b**) Change in H_2_O_2_ yield of Co-NC@CNT, Ni-NC@CNT, and CSCNT as a function of potentials.

Figure 7 shows the potential dependence on the electron transfer number and hydrogen peroxide yield obtained from LSV measurements. The ORR can proceed via two reaction pathways: the two- and four-electron reaction pathways. The four-electron reaction pathway is preferred, as it allows for the extraction of a larger number of electrons. The two-electron reaction pathway is not desirable, as it generates hydrogen peroxide, which negatively affects the catalytic property and the performance of LABs. The electron transfer number in Ni-NC@CNT, which was comparable to that in CSCNT, was below 3, indicating the dominance of the two-electron reaction type. Additionally, the hydrogen peroxide yield of Ni-NC@CNT was high, at approximately 70% at around 0.6 V, but decreased to approximately 50% as the potential decreased (i.e., a large overpotential). On the other hand, the electron transfer number in Co-NC@CNT was greater than 3, indicating the dominance of the four-electron reaction type.

Additionally, the hydrogen peroxide yield of Co-NC@CNT was lower than that of Ni-NC@CNT. These results demonstrate that the catalytic performance of Ni-NC@CNT for the ORR was slightly enhanced relative to that of CSCNT, whereas the catalytic performance of Co-NC@CNT was greatly improved. This enhancement arose because the SP treatment of CSCNT forms a composite with the N-containing carbon, resulting in the appearance of bond species with high ORR catalytic ability, such as pyridinic N, graphitic N, and Fe-N [8]. It has been reported that Co-N bonds and that composite materials of Co oxide and carbon exhibit superior ORR performance [37,38,39,40]. Therefore, in the case of Co-NC@CNT, the amorphous Co products observed by TEM may contribute to improving the ORR performance, but further studies are required to clarify this. 

**Figure 8:**
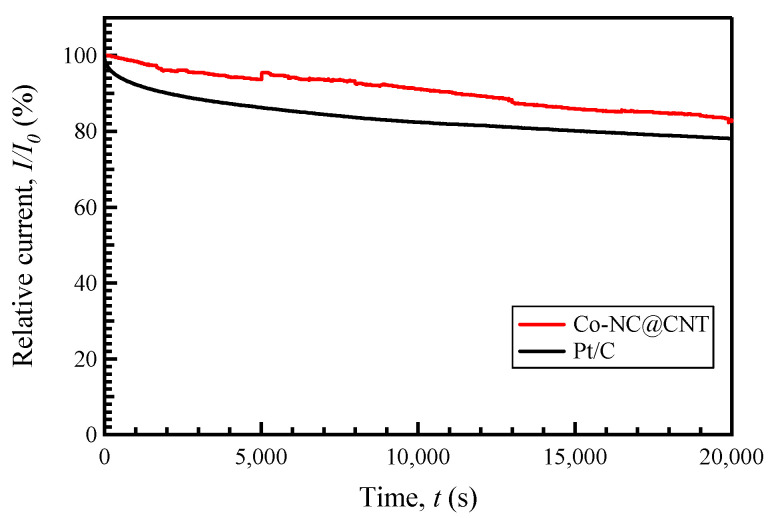
Chronoamperometric responses of Co-NC@CNT and Pt/C in O_2_-saturated 0.1 M KOH.

The current–time chronoamperometric (CA) response was measured at a constant potential of 0.45 V for 20,000 s in O_2_-saturated 0.1 M KOH (1600 rpm) to assess the durability of the most active Co-NC@CNT and 20% Pt/C, as shown in Figure 8. The relative current (*I*/*I*_0_, where *I*_0_ is the current density at the start of the measurement, and *I* is the current density at a specific time) of Co-NC@CNT was lower than the attenuation of the relative current of Pt/C. Thus, SP-treated Co-NC@CNT had higher long-term stability for the ORR than Pt/C, showing that Co-NC@CNT had superior durability compared to that of Pt/C, which is important for usage as the cathode material in LABs.

## 4. Conclusions

Transition metals Co and Ni were used as electrodes for the SP process and subjected to SP treatment in NMP containing FePc in which CSCNTs were dispersed. Through this process, we successfully synthesized composite materials of CSCNTs and carbon-containing Fe-N bonds, electrode-derived metals, and N. The N-doped carbon in the composite was amorphous and contained metal atoms, such as Fe, Co, and Ni. The carbon material subjected to the SP treatment using a Co electrode had a higher N content than the carbon treated with a Ni electrode. Moreover, the former had higher abundance ratios of Fe-N and graphitic N, which are chemical bonding species that effectively improve the catalytic performance and have a large specific surface area. The carbon material subjected to the SP treatment using the Co electrode exhibited superior ORR performance compared to the carbon treated with the Ni electrode. In addition, SP-treated Co-NC@CNT had higher long-term stability for the ORR than Pt/C, showing that Co-NC@CNT had superior durability compared to that of Pt/C.

## Figures and Tables

**Table 1:** Atomic composition ratio of the sample surfaces obtained by XPS (at%).

	C	N	O	Fe	Co	Ni
Co-NC@CNT	91.2	0.8	7.7	0.1	0.2	-
Ni-NC@CNT	89.6	0.7	9.5	0.1	-	0.1

**Table 2:** The relative component ratio of N-bonding species was obtained through the deconvolution of XPS N1*s* spectra. (at%).

	Pyridinic-N	Fe-N	Pyrrolic N	Graphitic N
Co-NC@CNT	34.5	3.3	33.9	28.3
Ni-NC@CNT	32.8	2.6	44.1	20.6

## Data Availability

Data are contained within the article and Supplementary Materials.

## Supplementary Materials

The following supporting information can be downloaded at: https://www.mdpi.com/article/10.3390/ma17020320/s1, Table S1. Comparison of ORR performance with other carbon materials. Refs. [6,8,10,12,13,20,22,23,29,35] are cited in the Supplementary Materials.
