# Supplementary material for: Solution-Plasma Synthesis and Characterization of Transition Metals and N-Containing Carbon–Carbon Nanotube Composites
Source: Materials (Basel). 2024 Jan 8;17(2):320. doi: 10.3390/ma17020320 (PMC10817228; doi:10.3390/ma17020320)
Supplement: Supplementary file 1 [file materials-17-00320-s001.zip › materials-2768809-supplementary.pdf]

materials

MDPI

Supplementary Materials

Table S1. Comparison of ORR performance with other carbon materials.

Catalyst	Doped element	Onset potential (V)	Half-wave potential (V)	Reference
Co-NC@CNT	N - 0.8 at%<br>Fe - 0.1 at%<br>Co - 0.2 at%	0.783	0.693	This study
Ni-NC@CNT	N - 0.7 at%<br>Fe - 0.1 at%<br>Co - 0.1 at%	0.749	0.686	This study
FePc–Py–CNTs			0.915	1
Fe–N-CNP–CNF	N - 1.16 at%<br>Fe - 0.15 at%	0.864		2
FeNI-CNS	N - 1.18 at%<br>I - 4.10 at%<br>Fe - 0.22 at%	0.968	0.847	3
Fe/N-CNT	N - 8.42 at%<br>Fe - 1.57 at%	0.96	0.81	4
Co/N-CNT	N - 8.36 at%<br>Co - 1.63 at%	0.94	0.84	4
Ni/N-CNT	N - 8.49 at%<br>Ni - 1.73 at%	0.91	0.73	4
NCNP-CNF	N - 1.35 at%	0.824		5
NGS	N - 6.00 at%	0.784	0.684	6
NCNS	N - 1.30 at%	0.794		7
FP-NCNs-SP	N - 2.3 at%<br>Fe - <0.1 at%	0.902		8
Co@N/GNP		0.98	0.87	9
rGO		0.77	0.65	10
NrGO800	N - 12.9 at%	0.88	0.76	10
CSCNT		0.749	0.667	This study
Pt/C	Pt - 20 wt.%	0.954	0.86	This study

References

1. Cao, R.; Thapa, R.; Kim, H.; Xu, X.; Kim, M. G.; Li, Q.; Park, N.; Liu, M.; Cho, J. Promotion of oxygen reduction by a bio-inspired tethered iron phthalocyanine carbon nanotube-based catalyst. *Nat. Commun.* **2013**, *4*, 2076. [CrossRef]
2. Panomsuwan, G.; Saito, N.; Ishizaki, T. Fe–N-doped carbon-based composite as an efficient and durable electrocatalyst for the oxygen reduction reaction. *RSC Adv.* **2016**, *6*, 114553–114559. [CrossRef]
3. Kim, H.; Cha, B.; Kim, D., Simultaneous introduction of iodine and Fe-N_x into carbon nanospheres for enhanced catalytic activity towards oxygen reduction using a solution plasma process. *Electrochem. Commun*. **2023**, *156*, 107589. [CrossRef]
4. Liu, Y.; Jiang, H.; Zhu, Y.; Yang, X.; Li, C. Transition metals (Fe, Co, and Ni) encapsulated in nitrogen-doped carbon nanotubes as bi-functional catalysts for oxygen electrode reactions. *J. Mater. Chem. A.* **2016**, *4*, 1694–1701. [CrossRef]
5. Panomsuwan, G.; Saito, N.; Ishizaki, T. Nitrogen-Doped Carbon Nanoparticle–Carbon Nanofiber Composite as an Efficient Metal-Free Cathode Catalyst for Oxygen Reduction Reaction. *ACS Appl. Mater. Interfaces* **2016**, *8*, 6962–6971 (2016). [CrossRef]
6. Lee, S.; Saito, N., Enhancement of nitrogen self-doped nanocarbons electrocatalyst via tune-up solution plasma synthesis. *RSC Adv*. **2018**, *8*, 35503–35511. [CrossRef]
7. Hyun, K.; Ueno, T.; Li, O.L.; Saito, N. Synthesis of heteroatom-carbon nanosheets by solution plasma processing using N-methyl-2-pyrrolidone as precursor. *RSC Adv.* **2016**, *6*, 6990–6996. [CrossRef]
8. Hyun, K.; Ueno, T.; Panomsuwan, G.; Li, O.L.; Saito, N. Heterocarbon nanosheets incorporating iron phthalocyanine for oxygen reduction reaction in both alkaline and acidic media. *Phys. Chem. Chem. Phys*. **2016**, *18*, 10856–10863. [CrossRef]
9. Jeong, S.; Kim, S.; Son, H.; Li, O. L., Plasma-engineered cobalt nanoparticle encapsulated N-doped graphene nanoplatelets as high-performance oxygen reduction reaction electrocatalysts for aluminum–air batteries. *Catal. Today* **2023**, *420*, 114025. [CrossRef]
10. Lemes, G.; Sebastian, D.; Pastor, E.; Lazaro, J. M. N-doped graphene catalysts with high nitrogen concentration for the oxygen reduction reaction. *J. Power Sources*. **2019**, *438*, 227036. [CrossRef]

Disclaimer/Publisher's Note: The statements, opinions and data contained in all publications are solely those of the individual author(s) and contributor(s) and not of MDPI and/or the editor(s). MDPI and/or the editor(s) disclaim responsibility for any injury to people or property resulting from any ideas, methods, instructions or products referred to in the content.
